# Commentary: Association between *Helicobacter pylori* infection and metabolic syndrome and its components

**DOI:** 10.3389/fendo.2023.1270855

**Published:** 2023-08-21

**Authors:** Toshihiko Kakiuchi

**Affiliations:** Department of Pediatrics, Faculty of Medicine, Saga University, Saga, Japan

**Keywords:** *Helicobacter pylori*, metabolic syndrome, child, obesity, *Prevotella* genus

## 1 Introduction

Liu et al. (1) published “Association between *Helicobacter pylori* (*H. pylori*) infection and metabolic syndrome (MetS) and its components,” suggesting that *H. pylori* infection is associated with MetS in males. Males and females in the *H. pylori*-infected group had a significantly higher prevalence of obesity, one of the components of MetS, than those in the uninfected group. Most studies of adults from different countries and regions have demonstrated a positive correlation between *H. pylori* infection and MetS (2). However, although studies have evaluated the relationship between *H. pylori* infection and body weight in children (3), no studies have directly investigated the relationship between *H. pylori* infection and MetS.

*H. pylori* infection, an established cause of gastritis, peptic ulcer, and gastric cancer, has an estimated worldwide prevalence of 50% (4) and 80%–90% among adults and adolescents in developing countries, respectively (5, 6). Although pediatric obesity has become an international problem (7) and the acquisition of *H. pylori* infection frequently occurs in childhood (8), a major problem is the lack of research on the association between *H. pylori* and MetS.

## 2 Association between MetS and *H. pylori* in children

### 2.1 Body height and weight of children undergoing *H. pylori* eradication treatment

An *H. pylori* testing and treatment strategy for children and adolescents was reported in Japan (9). Since 2016, we have been implementing a testing and treatment program for *H. pylori* among third-year junior high school students in Saga Prefecture (10, 11). The pediatric guidelines from several learned societies on the management of *H. pylori* in children have recommended against a “testing and treatment” strategy for *H. pylori* (12). Conversely, the guidelines have also added that in regions with high rates of gastric cancer including China and Japan, the benefits of treatments that reduce the risk of gastric cancer development may outweigh the risks of treatment. In Japan, where gastric cancer incidence is high, the *H. pylori* testing and treatment strategy for children and adolescents is implemented in several local governments. Between 2016 and 2022, 126 students underwent eradication treatment at the age of 15 at our hospital, of whom 91 (males, 50; females, 41) had their body height and weight measured. The average height and weight of male students were 166.92 ± 6.09 cm and 58.83 ± 11.51 kg, respectively. The average height and weight of female students were 156.23 ± 7.34 cm and 52.05 ± 9.40 kg, respectively. These results were compared with those of the annual report of school health statistics research compiled by the Ministry of Education, Culture, Sports, Science and Technology of Japan for male and female students, and no differences were observed. The results of this report were the average data of 57,966 individuals, accounting for 5.3% of 15-year-old individuals in Japan in 2021.

### 2.2 Analysis of the intestinal microbiota of children with *H. pylori* infection

**Figure 1:**
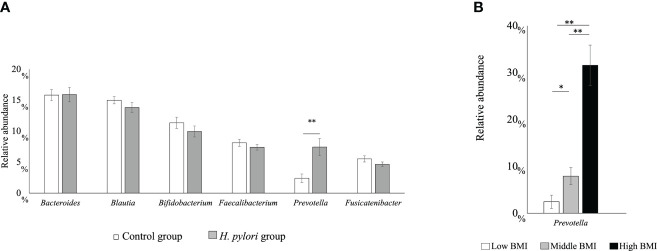
**(A)** The six main bacterial types present in the intestinal microbiota at the genus level. Comparison between the *H pylori*-infected and control groups (**p < 0.01). **(B)** Relative abundance of the *Prevotella* genus with regard to the body mass index (BMI) category in the *H pylori*-infected and control groups (*p < 0.05; **p < 0.01; ***p < 0.001). Low BMI, <15 kg/m^2^; mid BMI, 15–25 kg/m^2^; high BMI, >25 kg/m^2^.

We analyzed the effects of *H. pylori* infection on the intestinal microbiota of children (13). This study included 80 *H. pylori*-infected and 79 noninfected 15-year-old adolescents. No significant differences could be observed in sex, age, and body mass index (BMI) between the groups. *H. pylori* infection-associated symptoms including abdominal symptoms were not evaluated in both groups. Intestinal microbiota samples were collected from feces. The *H. pylori*-infected group had a significantly higher relative prevalence of the *Prevotella* genus than the uninfected group (p < 0.01) (**Figure 1A**). Furthermore, the relative occupancy of the *Prevotella* genus in the *H. pylori*-infected group increased in proportion to the BMI (**Figure 1B**). Thus, *H. pylori* infection had a significant impact on the intestinal microbiota of Japanese adolescents.

## 3 Discussion

Currently, consistent diagnostic criteria for defining MetS in childhood and adolescence are not available. Since 1980, the prevalence of childhood obesity has more than doubled, and 6%–39% of obese children and adolescents already present with MetS, depending on the definition applied (14). The lack of diagnostic criteria for MetS in children is a major impediment to progress in research on the relationship between *H. pylori* and MetS.

Previous studies have evaluated the relationship between *H. pylori* infection and body weight in adults. Furthermore, this relationship has been evaluated in children, with various results reported including positive (15), negative (16–19), and no correlations (20). Our data did not differ when compared with randomized data from Japan (the annual report of school health statistics research). Previously, the *H. pylori* infection rate among 15-year-old individuals in Japan was approximately 2% (11). Although there may be various biases, in general, *H. pylori* infection seems to lead to poor weight gain in children. This is contrary to the findings of Liu et al., where *H. pylori* was associated with obesity, one of the components of MetS in adults. However, *H. pylori* eradication was reported to improve the nutritional status and weight gain in children (18, 20). The reasons for these differences in *H. pylori* and body weight between children and adults are unclear. As one of the reasons, pediatric studies on changes in weight come from studies on symptomatic pediatric patients, wherein ruling out the restriction of intakes owing to pain, dyspeptic symptoms, or adverse effects of any *H. pylori* eradication treatment is not possible. Regarding the relationship between *H. pylori* and MetS in children, whether the results are similar to those in adults is an issue that requires further investigation.

We compared the fecal intestinal microbiota of *H. pylori*-infected and noninfected children (13). Most studies on the effects of *H. pylori* on the intestinal microbiota based on the analysis of fecal samples were conducted in adults, and data from children are lacking (21). *H. pylori* infection in children with an increased BMI without diabetes mellitus, was associated with an increase in the prevalence of the *Prevotella* genus (22), which was concomitant with an increase in the BMI in our previous study (13) (**Figure 1B**). The prevalence of the *Prevotella* genus was increased in patients with obesity (23), nonalcoholic steatohepatitis (24), hyperlipidemia (25), and even in gestational diabetes, which is considered a diabetes mellitus preliminary group disease. It is believed that an increase in the *Prevotella* genus may be involved in abnormal glucose metabolism development as a result of obesity (26). Although there are no studies regarding an increase in the *Prevotella* genus in adults infected with *H. pylori*, the *Prevotella* genus might be increased owing to *H. pylori* infection, thereby resulting in MetS. This suggests that children infected with *H. pylori* are at risk of MetS.

## 4 Conclusion

To elucidate the relationship between *H. pylori* and MetS in children, developing international standards for MetS in children and clarifying the pathogenesis of *H. pylori*-induced MetS in adults are necessary.

## Author contributions

TK: Conceptualization, Supervision, Validation, Writing – original draft, Writing – review & editing.
